# Patient-Specific Electric Field Simulations and Acceleration Measurements for Objective Analysis of Intraoperative Stimulation Tests in the Thalamus

**DOI:** 10.3389/fnhum.2016.00577

**Published:** 2016-11-25

**Authors:** Simone Hemm, Daniela Pison, Fabiola Alonso, Ashesh Shah, Jérôme Coste, Jean-Jacques Lemaire, Karin Wårdell

**Affiliations:** ^1^Institute for Medical and Analytical Technologies, School of Life Sciences, University of Applied Sciences and Arts Northwestern Switzerland FHNWMuttenz, Switzerland; ^2^Department of Biomedical Engineering, Linköping UniversityLinköping, Sweden; ^3^Université Clermont Auvergne, Université d’Auvergne, EA 7282, Image Guided Clinical Neurosciences and Connectomics (IGCNC)Clermont-Ferrand, France; ^4^Service de Neurochirurgie, Hôpital Gabriel-Montpied, Centre Hospitalier Universitaire de Clermont-FerrandClermont-Ferrand, France

**Keywords:** deep brain stimulation (DBS), intraoperative stimulation tests, essential tremor, acceleration measurements, finite element method (FEM) simulations, ventral intermediate nucleus (VIM), patient-specific brain maps

## Abstract

Despite an increasing use of deep brain stimulation (DBS) the fundamental mechanisms of action remain largely unknown. Simulation of electric entities has previously been proposed for chronic DBS combined with subjective symptom evaluations, but not for intraoperative stimulation tests. The present paper introduces a method for an objective exploitation of intraoperative stimulation test data to identify the optimal implant position of the chronic DBS lead by relating the electric field (EF) simulations to the patient-specific anatomy and the clinical effects quantified by accelerometry. To illustrate the feasibility of this approach, it was applied to five patients with essential tremor bilaterally implanted in the ventral intermediate nucleus (VIM). The VIM and its neighborhood structures were preoperatively outlined in 3D on white matter attenuated inversion recovery MR images. Quantitative intraoperative clinical assessments were performed using accelerometry. EF simulations (*n* = 272) for intraoperative stimulation test data performed along two trajectories per side were set-up using the finite element method for 143 stimulation test positions. The resulting EF isosurface of 0.2 V/mm was superimposed to the outlined anatomical structures. The percentage of volume of each structure’s overlap was calculated and related to the corresponding clinical improvement. The proposed concept has been successfully applied to the five patients. For higher clinical improvements, not only the VIM but as well other neighboring structures were covered by the EF isosurfaces. The percentage of the volumes of the VIM, of the nucleus intermediate lateral of the thalamus and the prelemniscal radiations within the prerubral field of Forel increased for clinical improvements higher than 50% compared to improvements lower than 50%. The presented new concept allows a detailed and objective analysis of a high amount of intraoperative data to identify the optimal stimulation target. First results indicate agreement with published data hypothesizing that the stimulation of other structures than the VIM might be responsible for good clinical effects in essential tremor. (Clinical trial reference number: Ref: 2011-A00774-37/AU905)

## Introduction

Deep brain stimulation (DBS) is a common neurosurgical procedure for relieving movement disorders such as those observed in Parkinson’s disease (PD) (Benabid et al., 1993, 2009; Hemm and Wårdell, 2010), essential tremor (ET) (Benabid et al., 1991) and dystonia (Coubes et al., 2000; Cif et al., 2010). Despite an increasing use and an extension of the indications (Hariz et al., 2013), the fundamental mechanisms underlying stimulation-induced effects, either therapeutic or adverse, remain largely unknown. The exact anatomical regions or white matter fibers responsible for these effects are still subject of discussion (Herrington et al., 2015). During a typical surgical planning, the optimal implantation position for a specific target is first approached based on anatomical images. Intraoperatively, the micro contact of an exploration electrode is often used for micro-electrode recordings (MER) (Coste et al., 2009) to evaluate the neuronal activity at previously planned positions of deep brain structures. In a further step, intraoperative stimulation tests are performed through the macro contact of the exploration electrode at different locations with help of the MER-system, and changes in the patient’s symptoms are observed by clinical examination. The DBS electrode is finally implanted at the location with the highest therapeutic effect on the symptom with minimal stimulation amplitude and side effects, or with side effects occurring only for high stimulation amplitudes. This procedure is completely based on the physicians experience and will therefore vary depending on the clinical skills (Post et al., 2005).

A way to objectify this evaluation is to use accelerometer recordings of the movements. We have previously presented a method to support the physician’s evaluation during surgery by quantifying intraoperatively obtained therapeutic effects on tremor (Shah et al., 2016b) and rigidity (Shah et al., 2016a) with the help of wrist acceleration measurements. These results suggest that mathematical parameters extracted from the acceleration signal are more sensitive to detect changes in tremor during intraoperative stimulation tests than the subjective neurologist’s evaluation. An enhancement of this methodology would be to relate the wrist accelerometer measurements for the evaluation of intraoperative stimulation tests with the patient’s own brain anatomy and patient-specific simulations of the EF around the stimulation electrode.

The finite element method (FEM) is commonly used to simulate the distribution of the EF around DBS electrodes often taking into account the individual patient’s anatomical data (Åström et al., 2009; Chaturvedi et al., 2010; Wårdell et al., 2015). The established models have been applied to relate the results of long term chronic stimulation to anatomical structures surrounding the stimulating contact. However, the use of patient-specific models to simulate data acquired during intraoperative stimulation tests has not yet been proposed.

To support the patient-specific simulations and also the surgical planning, different brain atlases have been suggested over the years (Schaltenbrand and Bailey, 1959; Morel, 2007). This is especially important for brain nuclei generally not visible with current conventional magnetic resonance imaging (MRI). With specific sequences it is possible to detail most common substructures of the thalamus and of other deep brain regions (Zerroug et al., 2016). Lemaire et al. (2010) introduced a high resolution atlas of the thalamus which makes extraction of such nuclei possible.

The aim of the present study was to introduce a new methodology combining different patient-specific data to identify the optimal implant position of the chronic DBS lead: thalamic patient-specific brain maps, EF simulations for intraoperative stimulation tests based on patient-specific simulation models and the corresponding therapeutic effects quantitatively evaluated by wrist accelerometer recordings. To illustrate the feasibility of this methodology, it was applied to five patients with ET who underwent stimulation tests during targeting of the ventral intermediate nucleus of the thalamus (VIM). An exemplary way of analysis is presented by comparing the extension of stimulation for no/low and intermediate/high improvements.

## Materials and Methods

**FIGURE 1:**
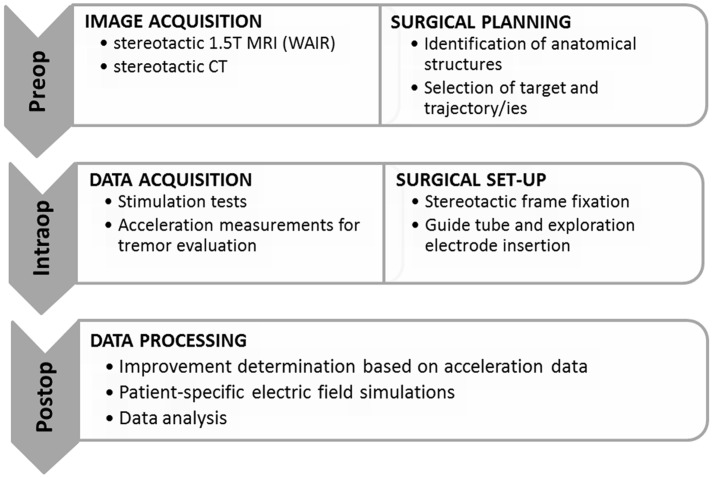
**Overview of the surgical workflow and the different data acquisition methods.** WAIR, white matter attenuated inversion recovery.

An overview of the methodology including imaging, generation of patient-specific maps of the thalamic region, surgical planning, surgical procedure, stimulation tests, accelerometer measurements, patient-specific EF simulations and data analysis is presented in **Figure 1**.

### Surgical Protocol

Stereotactic exploration and lead implantation were performed at the Department of Neurosurgery, Clermont-Ferrand University Hospital, France, under local anesthesia in a two-day procedure.

**FIGURE 2:**
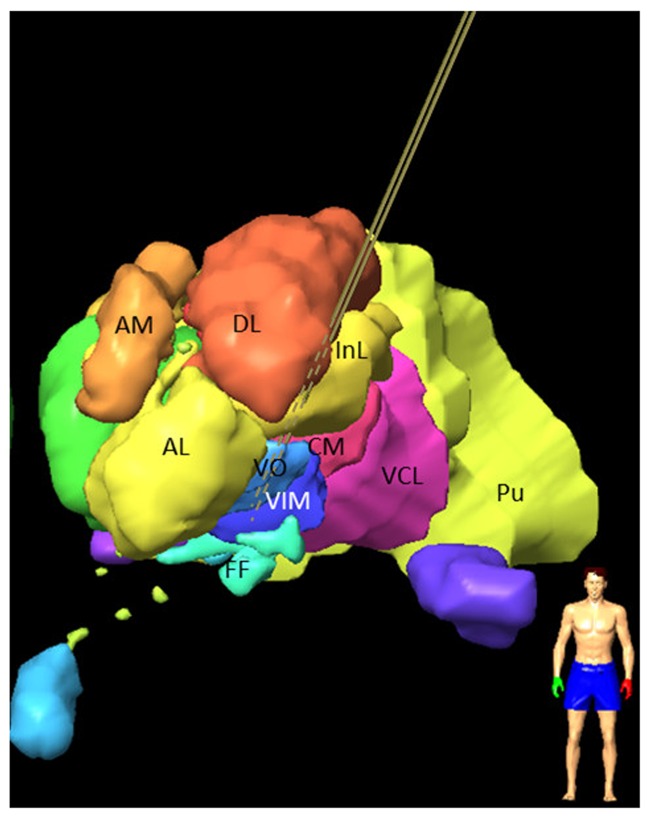
**Frontal view of left hemisphere of a 3D stereotactic planning for targeting the VIM, after manual outlining of the thalamic nuclei on 1.5 T WAIR images: VIM; VO; nucleus intermedio lateral (InL); nucleus ventrocaudal lateral (VCL); nucleus dorsolateral (DL); pulvinar (PU); nucleus anterolateral (AL); nucleus ventro-oral (VO); field of Forel (FF); nucleus centromedian (CM); the nucleus ventrocaudal medial (VCM) and the pre-lemniscal radiations (PLR) within the prerubral field of Forel are not visible.** Central and posterior left trajectories are visible (brown lines) and marked as dashed lines if inside the nuclei.

The first day, the stereotactic frame was mounted on the patient’s head (Leksell^®^ G frame, Elekta Instrument AB, Sweden) under local anesthesia. T1 MRI (0.63 mm × 0.63 mm × 1.30 mm) and white matter attenuated inversion recovery images (WAIR, 0.53 mm × 0.53 mm × 2.00 mm) (Magnotta et al., 2000; Lemaire et al., 2007) were acquired (Sonata, 1.5T, Siemens, Germany). Using a stereotactic planning software (iPlan 3, Brainlab, Feldkirchen, Germany), the VIM and its anatomic neighbors were carefully identified and manually outlined on the coronal plane of the WAIR sequence (Lemaire et al., 2010; Zerroug et al., 2016). The nuclei identification followed the previously published nomenclature (Lemaire et al., 2010; Vassal et al., 2012) based on their relative positions, intrinsic MRI tissue contrasts on 1.5T WAIR images (see Figure 6 in Zerroug et al., 2016) and an in-house microscopic 4.7T 3D T1 MRI atlas (see Figure 1 in Vassal et al., 2012). Target coordinates and two parallel trajectories were defined according to the stereotactic reference system, without AC-PC referencing. **Figure 2** shows a stereotactic planning including the patient-specific brain map and the planned trajectory.

The second day, after repositioning of the frame and stereotactic computed tomography (CT) acquisition (0.59 mm × 0.59 mm × 1.25 mm), the planned trajectories were checked and adjusted if necessary with the stereotactic reference system of the CT after rigid image fusion of WAIR and CT data sets. The target region was then explored intraoperatively (MicroGuide Pro; Alpha Omega Engineering, Nazareth, Israel) (Slavin and Burchiel, 2002) under local anesthesia using two exploration electrodes (Neuroprobe 366-000024, Alpha Omega Engineering, Nazareth, Israel) that were steered by rigid guide tubes (ACS-7905/200-5, DIXI Microtechniques, Besançon, France): one for the planned track (named the central track) and one placed 2 mm in parallel, usually posterior or posterolateral to the central one. MER was acquired in millimeter steps using the micro contact of the electrode which was retracted before starting stimulation tests in order to avoid tissue damage. Gradual stimulation tests were performed at the same locations through a macro contact to assess clinical benefit and adverse effects and to identify the optimal target. For each stimulation test, the surgical team identified and noted the maximum change in the patient’s tremor relative to the initial state of the patient (baseline), and the corresponding stimulation amplitude as well as the occurrence of side effects. MER and stimulation tests were in general performed in a range starting some millimeters in front of the target point and going slightly below depending on the anatomical location. In addition to this routine assessment of tremor, wrist accelerometer measurements and video recordings were performed. Following the stimulation tests, a quadripolar DBS-lead (Lead 3389, Medtronic Inc., USA) was implanted at the optimal stimulation spot for chronic stimulation.

### Acceleration Measurements

To perform intraoperative acceleration measurements, a 3-axis accelerometer, placed inside an in-house developed plastic case, was tied to the patient’s wrist on the opposite side of the stimulated hemisphere. Via a USB cable, the device was connected to a laptop based data recorder using homemade software LemurDBS (Java 1.6, Oracle, USA) (Shah et al., 2013). Synchronization between acceleration data and test stimulation amplitudes was assured by a pulse sent from the laptop to the stimulating equipment. The sensor was always attached at the same position on the wrist of the patient, and at each position a baseline recording was acquired before initiation of each stimulation sequence.

**FIGURE 3:**
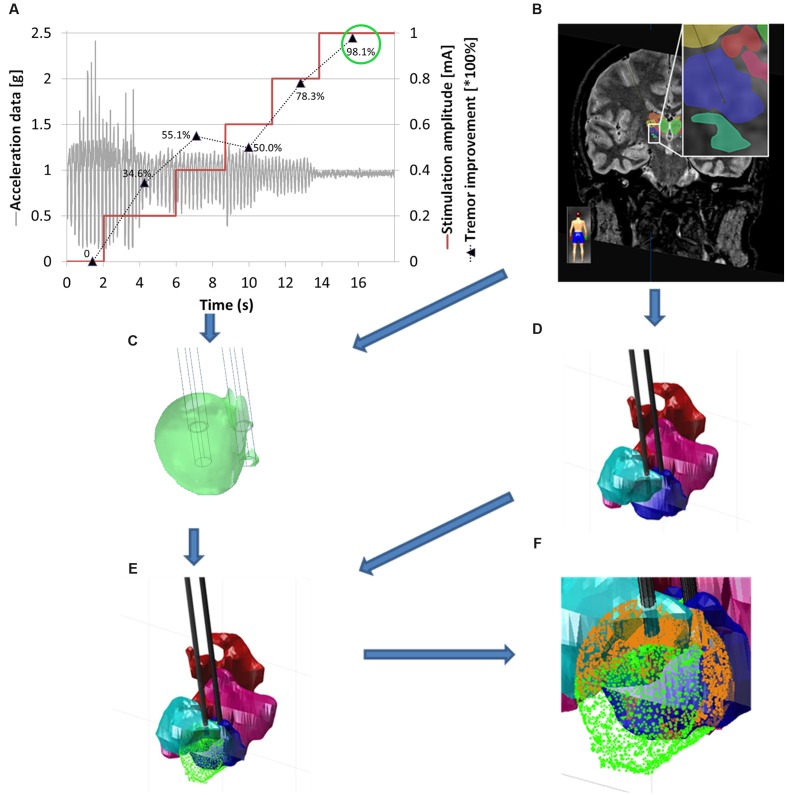
**Workflow for the generation of patient-specific 3D brain maps.** **(A)** Typical data in one stimulation position showing changes in tremor in relation to the increasing stimulation amplitude [mA] (red curve), filtered acceleration data [g] (gray curve), clinical improvement relative to baseline, quantified by acceleration measurements in percentage values from 0 to 100% (mean value for each stimulation amplitude; black dotted line). **(B)** MR-WAIR sequence used for planning including manually outlined structures. **(C)** Patient-specific EF simulation visualized at isolevel 0.2 V/mm at the stimulation position for an amplitude of 1 mA (green circle) corresponding to 98.1% improvement. **(D)** Manually outlined structures: CM (red), VO (light blue), VIM (dark blue) and VCM (pink). **(E)** Brain map with superimposed EF isosurface (green). **(F)** Close up view of **(E)** with the structure volumes inside the EF isosurface indicated in orange.

In order to quantify the clinical improvement for each stimulation amplitude, a previously developed analysis method in Matlab (R2014b) was used (Shah et al., 2016b). As a first step, movements other than tremor were removed offline by using the smoothness priors method (Tarvainen et al., 2002) and thereafter a second order Butterworth low pass-filter was applied at 10 Hz in order to suppress noise. Statistical features (standard deviation, signal energy and the spectral amplitude of the dominant frequency, defined as the frequency of the signal with maximum spectral power) were extracted by moving a 2 second-window over the data. These features were then normalized to the feature set representing the most intense tremor at baseline, i.e., during an initial off-stimulation period at the same position. For each stimulation amplitude the mean of the obtained quantitative clinical improvement was retained as a percentage value. An example is presented in **Figure 3A** where a typical acceleration signal in one position is presented together with the clinical improvement based on the calculated features. In the presented example, it can be seen that an improvement of 98.1% was obtained at a stimulation amplitude of 1.0 mA.

### Electric Field Simulations

In order to simulate the EF spatial distribution within the brain, a 3D FEM model of the exploration electrode with the surrounding brain tissue was built (Comsol Multiphysics, Version 4.4 Comsol AB, Sweden) for adapting an already established patient-specific modeling technique for DBS leads (Åström et al., 2010; Wårdell et al., 2015).

#### Brain Tissue Model

The axial preoperative T1 MRI was registered and resampled to the stereotactic preoperative axial CT dataset. In a next step, it was imported into the in-house developed software (Matlab R2013) (Wårdell et al., 2012) modified for the creation of the brain tissue models. A separate filtered axial T1 image batch with enhanced region of interest was used to segment cerebrospinal fluid (CSF), gray matter and white matter (Alonso et al., 2016). The segmented image voxels were assigned with the corresponding electrical conductivities (σ) (Gabriel et al., 1996)^1^. CSF and blood were set to 2.0 Siemens/meter (S/m) and 0.7 S/m, respectively. Considering the frequency (130 Hz) and pulse length (60 μs) of the stimulation (Wårdell et al., 2013), to gray matter was assigned 0.123 S/m and to white matter 0.075 S/m. Interpolation was done for conductivity values in-between the thresholds used. In order to reduce the simulation time, a region of interest (a cuboid of approximately 100 mm per side) covering the thalamus and its surroundings was selected from the brain tissue model.

#### Exploration Electrode and Guide Tube Model

**FIGURE 4:**
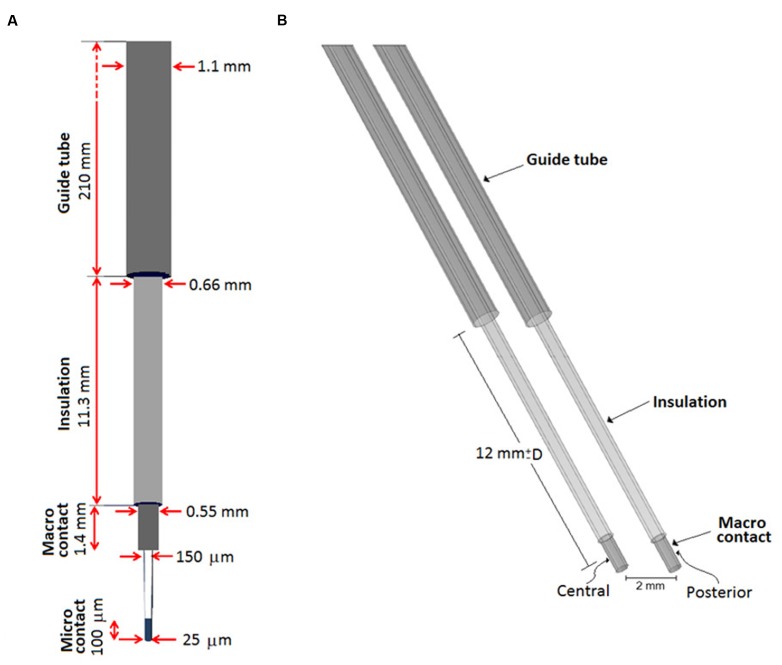
**(A)** Schematic representation of the guide tube and the exploration electrode including the micro contact for recording and the macro contact for stimulation, **(B)** FEM model of the exploration electrode (recording tip is excluded as it is retracted during stimulation) and the guide tube. The probe slides within the fixed grounded guide tube placed 12 mm from a target point corresponding to the *a priori* optimal anatomic spot. Explorations were performed moving the stimulation point along the trajectory, D millimeters proximal or distal to the target position.

A model of the stimulating contact of the exploration electrode and the guide tube was developed. **Figure 4A** presents the outer and inner dimensions of the exploration electrode and the guide tube, **Figure 4B** the corresponding model. The end of the guide tube was fixed 12 mm above the chosen target point, i.e., above the *a priori* optimal anatomic spot. A second exploration electrode and guide tube model was positioned in parallel at a distance of 2 mm. The distance between the guide tube and the center of the stimulating contact decreased or increased when the simulation site was ahead or beyond the target point, respectively. The center of the stimulating contact was placed at the different planned stimulation positions. The micro contact was not considered as it was retracted during the stimulation tests.

#### Simulations

The EF was calculated by using the equation of continuity for steady current according to:

$$\nabla \cdot \overset{¯}{j}=\nabla \cdot (\sigma \nabla V)=0$$

where *J* is the current density, σ a matrix containing the electrical conductivity values for the region of interest (thalamus and neighborhood) and *V* the electric potential. A monopolar configuration was conducted using the guide tube as the reference electrode setting it to ground, and the active electrode set to the same current as used during the stimulation tests. The non-active contact of the parallel lead was set to floating potential (Schmidt et al., 2013). The exterior boundaries of the tissue model were set to electrical insulation. The mesh density (consisting of about 250,000 tetrahedral elements) was defined by the built in physics-controlled mesh generator, where the smallest elements (0.204 mm) were located nearby the stimulating contacts in order to capture the strong EF gradients. The Cartesian coordinates of the points describing the surface of the simulated EF volume (**Figure 3C**) were exported for further analysis. In this study an EF isolevel of 0.2 V/mm was used in order to be able to perform relative comparisons between the simulations and to comply with approximate axon diameters in the thalamus (Kuncel et al., 2008; Åström et al., 2015; Alonso et al., 2016).

### Thalamic Brain Maps and Electric Field Visualization

The thalamic structures (**Figures 2** and **3B**) initially outlined on the WAIR weighted sequence in the iPlan software were exported in form of slices parallel to the stereotactic CT data set via an interface based on VVLink and VTK (VTK 5.2.0, Kitware Inc., Clifton Park, NY, USA). Target and trajectory coordinates were also exported in CT image coordinates by the same software interface. The CT data set was chosen as it provides a higher resolution and no distortion of the stereotactic reference system compared to MR sequences. With the exported data a 3D thalamic brain map with trajectories was generated in Matlab (R2014b) (**Figure 3D**). For each stimulation test position and amplitude, the EF isosurface generated through FEM simulations was imported, superimposed to the 3D thalamic brain map and color-coded depending on the induced, quantitatively evaluated improvement (**Figure 3E**).

### Volumetric Analysis

An in-house algorithm developed by FHNW in Matlab (R2014b) was applied to detect and calculate the volume of the anatomical structures inside each EF isosurface. To reduce the computational time, a list of candidate structures (e.g., VIM, VO, and CM) was identified from the entire structure group by excluding the structures outside the coordinate’s ranges of the EF volume. For each candidate structure, the points of the EF isosurface inside the structures’ volume were detected by considering them as a concave or convex hull according to their shape. The obtained volume based on the selected point cloud was then calculated and associated with the respective clinical improvement. The algorithms then generated a list of the thalamic structures lying partially or completely inside the 0.2 V/mm EF isosurface, their volumes as well as the volume covered by the EF surface and the associated improvement value (**Figure 3F**).

### Clinical Application

The above presented protocol was applied to five ET patients (three male and two female) undergoing bilateral DBS electrode implantation in the VIM region and successively to both hemispheres. They gave their written informed consent to participate in the study (Ref: 2011-A00774-37/AU905, Comité de Protection des Personnes Sud-Est 6, Clermont-Ferrand, France). No alterations were made to the routine surgical procedure. In all patients a central and a posterior trajectory were chosen per hemisphere for MER and stimulation tests (stimulation parameters: amplitude = 0.2 to 3.0 mA in steps of 0.2 mA, pulse width = 60 μs, frequency = 130 Hz). At each stimulation position stimulation lasted 1 to 3 min depending on the response of the patient and on side effect occurrence or not. Between all stimulation tests, a non-stimulation period was maintained to leave time to the symptoms to come back. The duration of this period depended on patient symptoms (minimum 2 min). Acceleration measurements were performed in parallel to the test stimulation in 31, 22, 30, 28, and 32 positions for Patient 1 to 5, respectively, mostly from 5 mm above the target point down to 4 mm below depending on the individual anatomical locations. The final electrode implantation site was based on clinical subjective evaluations.

**FIGURE 5:**
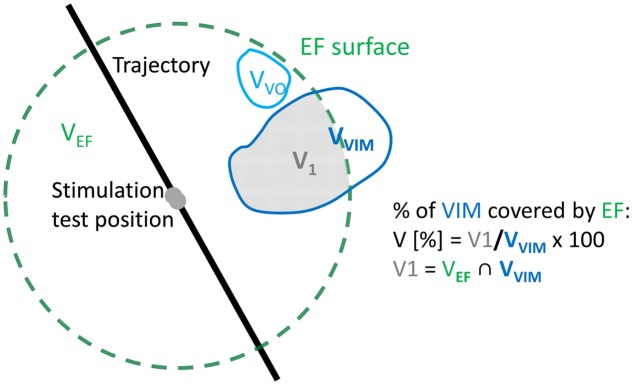
**Visual example of the calculation of the percentage of volume of VIM (gray area) inside the EF (dashed green line).** The volume V_1_ is the intersection between the volume of the EF (V*_EF_*) and the volume of VIM (V*_VIM_*).

Electric field simulations were performed for all stimulation test positions in both hemispheres of the five patients. At each position, up to four tested stimulation amplitudes were chosen for simulations using the following criteria based on the quantitatively evaluated symptom improvements (*I*_acc_): (1) The highest amplitude not resulting in any improvement in tremor compared to baseline; (2) the lowest amplitude at which a first improvement in tremor was measured; (3) The lowest amplitude resulting in at least 50% improvement in tremor; (4) The lowest amplitude resulting in at least 75% improvement. When the first improvement in tremor was more than 75%, criteria (2–4) gave the same amplitude. When the first improvement was identified already between 0.2 and 0.6 mA, no simulations were performed for the criterion 1. The extracted patient-specific 3D brain maps of the thalamus were superimposed with the four trajectories of each patient and with the simulated patient-specific EF isosurfaces. To make the data comparable between patients, the volume inside the isosurface was normalized to the size of the structure resulting in the percentage of the structure covered by the EF (**Figure 5**). For example, if the volume of VIM was 10 mm^3^ and only 2 mm^3^ of it was encompassed by an EF isosurface, the covered volume of VIM for that EF would have been (2/10)^∗^100 = 20%.

In order to identify structures responsible for the reduction in tremor, the results of all patients together were classified following the quantity of improvement detected by accelerometry (*I*acc). Data were divided into two groups considering no/low improvements (*I*_acc_ ≤ 50%) and intermediate/high improvements (*I*_acc_ > 50%), respectively. The resulting data are presented in two different ways for comparison of these two improvement groups. Firstly, for each thalamic structure, the relative number of occurrences (the structure is at least partially covered by the EF) was determined: the absolute number of occurrences of *each structure* in the considered improvement range was normalized to the total number of occurrences of *all structures* in this range. Second, the percentage volume of each structure covered by a specific EF isosurface was analyzed to see for example if the covered volume of some structures increases for higher improvements. These percentage volumes were graphically represented and visually analyzed together with the induced clinical improvement for all simulations. Furthermore, mean values and the standard error of the mean (SEM) were determined for each structure for the two improvement groups. The results for each structure in the two improvement ranges were statistically compared applying the Mann–Whitney *U* test. Mean stimulation amplitudes for 50% or less improvement and more than 50% improvement were determined.

## Results

### Simulations

The proposed concept has been successfully applied to the five patients, resulting in 272 simulations at 143 different stimulation test positions. The detailed numbers of simulations for each patient and different improvement ranges are presented in **Table 1**.

**Table 1:** Number of simulations per patient and clinical improvement range as recorded by accelerometry (*I*_acc_).

	0 < *I*_acc_ ≤ 25%	25 < *I*_acc_ ≤ 50%	50 < *I*_acc_ ≤ 75%	75% < *I*_acc_ ≤ 100%	Total
Patient 1	3	13	17	12	45
Patient 2	3	11	20	10	44
Patient 3	1	23	15	22	61
Patient 4	2	9	23	18	52
Patient 5	2	15	23	30	70
Total	11	71	98	92	272


### Visualization

**FIGURE 6:**
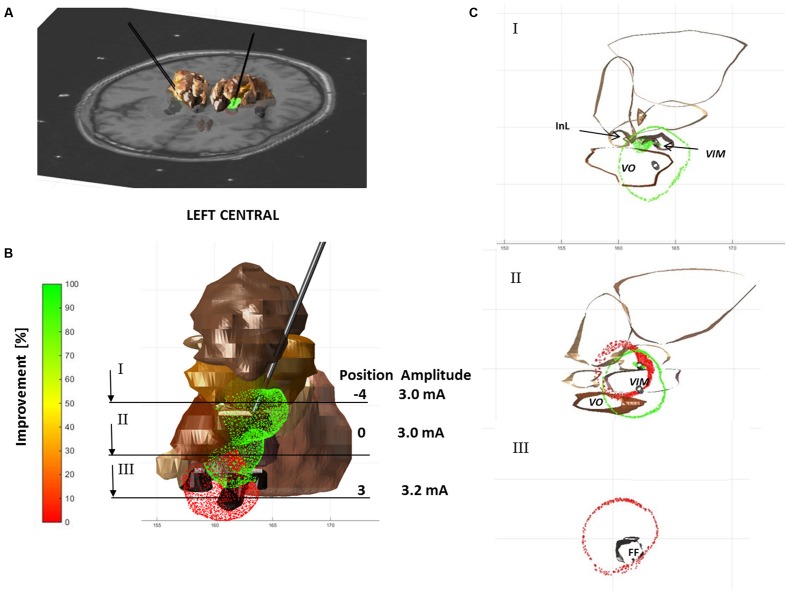
**EF isosurfaces for Patient 4 simulated for three positions (proximal, zero, and distal) on the central electrode track of the left hemisphere, color-coded following the induced clinical change (red – 0% and green – 100% improvement) and superimposed to selected anatomical structures (brown).** **(A)** Axial MRI superimposed to 3D structures and EF isosurfaces. **(B)** 3D frontal view of structures and electric fields; positions and amplitude of the simulations are summarized below; lines indicate the positions of sections visualized in **(C)**. **(C)** Three axial sections at different levels: proximal (negative numbers), at (0) and distal (positive numbers) to the target.

**Figure 6** shows an example of visualization for Patient 4 with three simulated EF isosurfaces in the left hemisphere along the central tract. Each isolevel is superimposed to the extracted anatomical structures (seen in brown) and the patient-specific MRI (**Figure 6A**). At the target position or the *a priori* optimal anatomic spot for the left central trajectory an improvement of 90% was reached with a stimulation amplitude of 3 mA (**Figure 6B**). 3 mm below the target no improvement in tremor could be observed. The corresponding EF in red overlays the EF of 90% of improvement as can be seen at cross section II through the stimulation electrode as presented in **Figure 6C**.

### Involvement of Anatomical Structures

**FIGURE 7:**
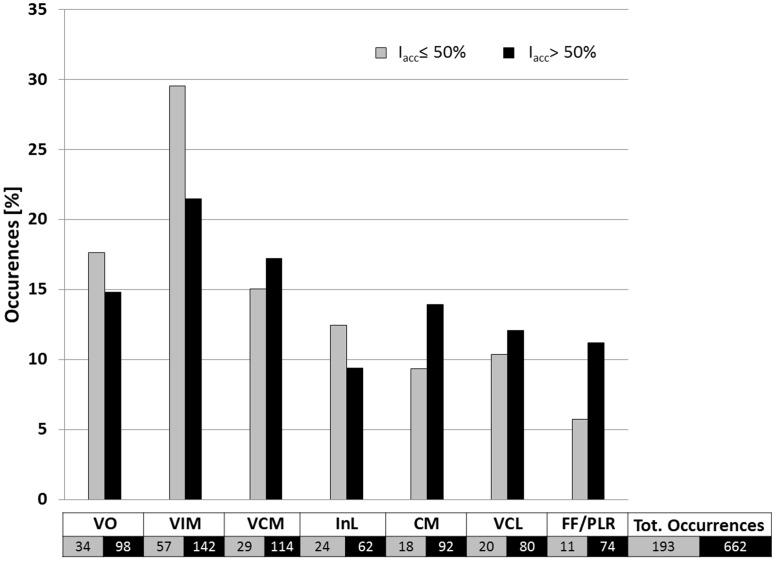
**Occurrence of anatomical structures related to improvements of *I*_acc_ ≤ 50% (gray bars; *n* = 193) and of *I*_acc_ > 50% (black bars; *n* = 662) in percent of the total number of structures occurring inside electrical field isosurfaces of 0.2 V/mm.** For structure nomenclature, see **Figure 2**.

The relative occurrences of the different thalamic structures within the isosurfaces for improvements above and below 50% are presented in **Figure 7**. It shows that the percentage of occurrences of the different structures was always inferior or equal to 30%. The relative occurrence of InL, VO and especially VIM decreases for higher improvements. This means that their appearance does not as much increase as for CM, VCM, VCL and especially for FF/PLR. For all four structures the relative occurrence increases for higher improvements. Mean stimulation amplitudes for improvements *I*_acc_ ≤ 50% and *I*_acc_ > 50% were 0.9 ± 1.1 mA and 1.5 ± 1.2 mA, respectively.

### Relation of Structure Occurrences, Clinical Improvement, and Volumes Covered by the Isosurfaces

**FIGURE 8:**
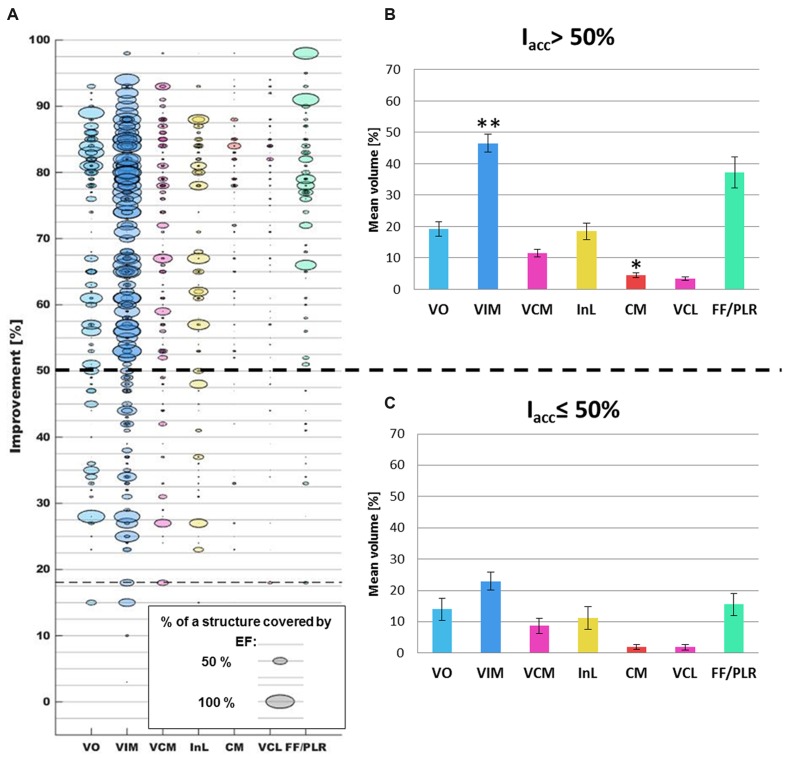
**(A)** Comparison of the occurrences of the different thalamic brain structures (*x*-axis; each color represents one structure) with the corresponding clinical improvement evaluated by accelerometry (*y*-axis) for all simulations (*n* = 272). Structures appearing in a same simulation have identical clinical improvement and can in consequence be found on the same horizontal level. As an example, the horizontal line at an improvement of approximately 18% indicates that VIM, VCM, VCL, and FF/PLR were present inside a same EF isosurface. **(B)** Summary of the mean percentage volume included in the EF isosurfaces and the standard error of the mean for the different structures in the improvement range >50% and **(C)** in the improvement range ≤50%. ^∗∗^: Statistically significant difference between the results of the two improvement groups for a specific structure with *p* < 0.001. ^∗^: Statistically significant difference with *p* < 0.01.

A comparison between the clinical improvement and the volume of the structures included in the isosurface of the corresponding simulation is presented in **Figure 8**. While **Figure 8A** shows all available data of the five patients, **Figures 8B,C** summarize the data in form of mean and SEM for improvements ≤50% and >50%. All SEM values remain below 3% except for the VO and the InL for the range *I*_acc_ ≤ 50% and the FF/PLR for both improvement ranges. A closer analysis of the volume of the different structures covered by the EF isosurface shows that the percentage volumes of the target structure VIM, of the InL and of the FF/PLR increase with significant clinical improvements. The difference for the VIM was statistically significant (*p* < 0001). Only small volumes of CM and VCL are covered by the isosurface in both improvement ranges. Nevertheless the difference for the CM could be shown to be significant (*p* < 0.01). The neighboring nuclei VIM and VCM appear together for nearly all simulations. FF/PLR and VO occur mostly in combination with VIM and VCM (same horizontal line) (**Figure 8A**).

## Discussion

In the present study, a methodology is described that has the potential to give new insights into the efficacy of different anatomical structures in DBS. It consists in the combined analysis of intraoperatively acquired accelerometry data, patient-specific EF simulations for intraoperative stimulation tests and patient-specific anatomy. The method was successfully applied to five patients with ET and included more than 250 EF simulations. An exemplary way of analysis and preliminary results have been presented for the identification of the therapeutically effective anatomical region.

### Quantitative Symptom Evaluation

In order to overcome the limits of existing routinely used clinical rating scales, i.e., the inter- and intra-observer variability (Post et al., 2005; Palmer et al., 2010), the discrete evaluation levels and the high dependence on the experience of the evaluating neurologist (Griffiths et al., 2012), we have used accelerometry-based, quantitative tremor evaluations during intraoperative stimulation tests.

Tremor quantification outside the OR has been proposed since a long time by various authors (Mansur et al., 2007), many of whom have concluded that a quantitative evaluation method is more sensitive than the visually performed clinical evaluation. Birdno et al. (2008) used an acceleration sensor to study the effects of temporal variations of the stimulation pulse during the replacement of the implantable pulse-generator. Journee et al. (2007) and Papapetropoulos et al. (2008) used quantitative tremor evaluation after the DBS lead was implanted, in order to compare the effects of stimulation through different contacts. But those systems were not designed to be used in different clinical centers or during stimulation tests performed through an exploration electrode. In a previous study, we have demonstrated the use of our system in 15 DBS surgeries in two different clinical centers, the possibility to visualize and revisit recorded data during surgery and the possible influences of quantitative evaluations on the choice of the final implant position of the lead for chronic stimulation (Shah et al., 2016b).

### Determination of the Therapeutically Implicated Structures

In the present clinical study, structures individually outlined by the neurosurgeon were available and could be used as anatomical reference. The use of the patient-specific MR-WAIR sequence together with a 4.7 T in-house atlas as reference and stereotactic books make an approximate identification of the structures possible (Zerroug et al., 2016). Other groups have proposed various approaches (Caire et al., 2013) among them projecting the position of the active contact(s) directly onto anatomical images (Vayssiere et al., 2004), onto anatomical (Saint-Cyr et al., 2002; Sarnthein et al., 2013) or probabilistic functional atlases (Lalys et al., 2013), or linking them to MER results (Zonenshayn et al., 2004) sometimes combined with imaging data (Weise et al., 2013) and white matter tracking (Coenen et al., 2012). To analyze the relationship between the anatomical location of stimulating contacts and the clinical effectiveness of stimulation, we have decided to take into account the extent of stimulation by using EF simulations (Åström et al., 2012) as discussed in detail in the next paragraph. Other published approaches consider either the anatomical position of the center of the contact (Starr et al., 2002; Voges et al., 2009; Garcia-Garcia et al., 2016) or of the whole contact taking into account its dimensions (Saint-Cyr et al., 2002; Zonenshayn et al., 2004; Herzog et al., 2007; Hemm et al., 2008).

### Electric Field Simulations

Finite element method models are commonly used to simulate and visualize the EF distribution around DBS electrodes and the EF is one of the electrical entities that may be used to represent the stimulation field. In comparison with the electric potential or the second derivative of the electric potential with respect to the distance (activating function), EF has been shown to be the most stable and unchanged entity for different stimulation parameters (amplitude and pulse width) (Åström et al., 2015).

Today, FEM models have progressed from non-specific (McIntyre et al., 2004; Hemm et al., 2005; Åström et al., 2006) to patient-specific taking into account the individual’s data (Åström et al., 2009; Chaturvedi et al., 2010; Wårdell et al., 2015). There is no consensus of the degree of complexity of the model to accurately simulate the neural response, however, many groups (Chaturvedi et al., 2010; Schmidt et al., 2013; Alonso et al., 2016; Howell and McIntyre, 2016) have shown that the inclusion of the heterogeneity and anisotropy of the brain tissue increases the model accuracy and prediction capability. For instance, Chaturvedi et al. (2010) and Åström et al. (2012) observed an overestimation of neural activation for homogeneous models. The present study relies on a brain model built upon the segmentation of the gray matter, white matter, CSF and blood from the patients’ MRI and in consequence takes into consideration the inhomogeneity of brain tissue. An even more realistic model may be based on DTI which provides more anisotropic information, however, its resolution is lower than the one of MRI and may introduce other errors (Åström et al., 2012). The simulations in this study were performed for constant current while the dispersive components of the brain tissue have been considered by adjusting the conductivity values for gray and white matter to the particular stimulation frequency and pulse width (Wårdell et al., 2013).

According to previous studies where neuron activation distances were calculated using neuronal models (Åström et al., 2015), an isolevel of 0.2 V/mm represents an equivalent activation distance for neurons within 3–4 μm of diameter (Alonso et al., 2016) and thus seems to comply with axon diameters in the thalamus as previously calculated by Åström et al. (Figure 6, 2015) based on Kuncel et al. (2008). The selection of a fixed EF isolevel allows then to compare the volume recruited for different amplitude settings and different positions.

### Transferability

The described methodology has been presented for an institution-specific surgical protocol but can be transferred to other clinical centers. The approach can be adapted to any kind of anatomical information. Instead of using manually outlined structures, it is possible to combine the generated data with anatomical atlases – with the limitations inherent to such an approach (Vayssiere et al., 2002; Wodarg et al., 2012; Anthofer et al., 2014) – or with fiber tracking data in order to analyze the implication of different fibers in the mechanism of action of DBS (Coenen et al., 2012). The MR image data (T1, T2) that are needed for the EF simulations are generally acquired in every institution for the surgical planning procedure. A modification of the developed model to the institution-specific stimulation test protocol in awake patients might be necessary: the characteristics of the stimulating electrode as well as the position of the guide tube during stimulation have to be adapted. The acceleration data recording can relatively easily be added in the intraoperative phase without any changes in the surgical protocol, without lengthening surgery and most importantly, without any discomfort for the patient. Nevertheless, the correlation of the simulation results can be performed as well based on subjective visual evaluations.

### Clinical Application

The results of the present paper are described as relative occurrences and percentage volumes of the different anatomical structures covered by the EF isosurface. Even if the number of patients presenting ET in our clinical study was low and thus the confidence concerning the analysis of the mechanism of action of VIM-DBS is limited, we can present preliminary results thanks to the high number of stimulation test positions and EF simulations per patient. First results of these EF simulations in **Figure 7** show that the percentage of occurrence of VCM, CM, and FF/PLR increases for higher improvements while the percentage of VIM occurrences decreases. This can be explained by the fact that in 60 out of 143 measurement sites the center of the stimulation contact was already within the VIM. Furthermore, as shown, there is a tendency that higher improvements are linked to higher stimulation amplitudes leading in general to a larger distribution of EF for a same tissue type. When looking at the percentage of volume of the VIM covered by the isosurface between the two improvement groups (**Figures 8B,C**), a statically significant difference exists. Nevertheless, the size of the individual volumes varies inside each group. This result can be interpreted in two ways: either (I) a specific part of the VIM, for example the efferent fibers, has to be stimulated or (II) other structures than the VIM might at least partially be responsible for the therapeutic effect. Following our preliminary results such structures could be the InL or especially the fiber tracks FF/PLR. This hypothesis would confirm previously published data: parts of the InL have been earlier mentioned for tremor reduction (Hirai and Jones, 1989) and several authors (Spiegelmann et al., 2006; Vassal et al., 2012) have reported that chronic stimulation of PLR works very well. Some authors (Caparros-Lefebvre et al., 1999; Vassal et al., 2012) already suggested that parts of the VO or of the zona incerta (Fytagoridis et al., 2012) could be appropriate targets as well. Recently Groppa et al. (2014) proposed the dentatothalamic tract as key therapeutic DBS target structure.

Following **Figure 7** our data suggest that parts of VCL and CM might be stimulated in some cases. However, **Figure 8** shows that the structure volumes included in the isosurface are below 5% in both improvement ranges. In order to avoid misinterpretation, either patient-specific improvement maps should be used for presentation or thresholds should be introduced to exclude insignificant volumes.

An optimal stimulation position and statistically significant clinical conclusions can only be provided after the analysis of more intraoperative data, the identification of occurring structure combinations and especially the side effect occurrences, which have a major influence on the choice of the final implant position of the chronic DBS lead.

### Limitations and Future Work

The suggested methodology allows a detailed interpretation of intraoperatively acquired data but one has to be aware of certain limitations. First of all, the substantial caveats of non-stimulation factors influencing tremor are undeniable and unfortunately inherent in the operating room conditions. Nevertheless, we have employed various signal analysis techniques to minimize the effect of such non-stimulation factors on the evaluation of tremor using accelerometer. Furthermore, the method was defined in a way trying to limit transformation and fusion errors as much as possible (Zrinzo, 2010). Nevertheless due to the available data, WAIR and T1 MRI data sets containing the anatomic information had to be fused to the stereotactic preoperative CT data set providing the reference for the targeting procedure. Concerning the position of the stimulating contact in relation to the structures, we assumed that the microelectrode was positioned exactly as planned. This seems to be a reasonable approach as the microelectrode was the first entering the brain, and it has been observed that brain shifts in the final electrode position and trajectory can appear when the exploration electrode is replaced by the DBS lead (unpublished data).

As the anatomical information is based on the structures manually outlined on the preoperative image data set, the approach does not consider the movement of the tissue due to the electrodes’ insertion or brain shift between implantation sides. On the other hand, the use of these preoperative image data sets is common in analysis and simulation methods. The limitations are acceptable as postoperative image data sets present disturbing artifacts around the implanted DBS leads, in the region of interest. To increase the power of the statistic test performed in the present study, more data should be acquired from further patients and included in the analysis. A further limitation, specific to the anatomical information, concerns the availability of only some anatomical structures and the FF/PLR and that always part of the volume of the EF isosurface is outside any manually defined anatomical structure. In consequence, information from white matter fiber tracking would be helpful to define the region anterior to the VIM and the InL and for further investigating possible activation of fiber tracks (Coenen et al., 2012).

The method could in a next step also be complemented with the available MER data at the different positions including the analysis of time patterns describing the network dynamics as proposed by Andres et al. (2015).

The data analysis approach proposed in the present paper considers the percentage of the structure volume covered by the simulated EF isosurface and not which parts of the structure. Further data interpretation could consider the 3D position as well and should generate improvement maps taking into account stimulation positions of amplitudes as well as the occurrence of side effects.

## Conclusion

A new concept for the analysis of data acquired during DBS surgery has been proposed. A workflow and methodology combining objective intraoperative tremor evaluation with patient-specific EF simulations on manually outlined anatomical structures has been defined and applied to five patients with ET undergoing DBS-implantation. This new approach is combined with an algorithm for detection of the volume of the anatomical structures involved during intraoperative microelectrode stimulation. It can be adapted to further surgical protocols, intraoperative set-ups and to other anatomical data. Its application will allow the analysis of intraoperative data obtained in clinical routine and will support the identification of anatomical structures, parts of them or white matter fibers responsible for the therapeutic effect. The analysis of more data and inclusion of occurrence of side effects are necessary to draw any final conclusions of the most efficient brain targets. The first results, however, indicate agreement with published data hypothesizing that the stimulation of structures other than the VIM might be responsible for good clinical effect in ET.

## Author Contributions

All authors contributed in writing the manuscript and critically reviewed the last version. SH: Idea and conception of whole approach; design of protocols and clinical study, especially acceleration measurements; set-up of whole method; development of brain map extraction; participation in data analysis and interpretation; main drafting of manuscript. DP: Technical set-up and implementation of the defined workflow; final data analysis of all available multimodal data; participation in data interpretation; main drafting of manuscript. FA: Set-up of the EF simulation with its patient-specific models; performing all the simulations; data analysis. AS: Realization of acceleration measurements and their analysis; choice of parameters for simulation. JC: Set-up of clinical study and intraoperative realization of study; stimulation data acquisition. J-JL: Set-up of clinical study; patient selection and operation; data interpretation. KW: Set-up of initial e-field simulations; conception of work from simulation point of view; support and critical review of whole concept and especially patient-specific simulations.

## Conflict of Interest Statement

The authors declare that the research was conducted in the absence of any commercial or financial relationships that could be construed as a potential conflict of interest.

## Footnotes

1 Andreuccetti, D., R. Fossi and C. Petrucci, Florence, Italy. (2005). “Dielectric properties of the tissue.” from http://niremf.ifac.cnr.it/tissprop/.
